# Marine *Rhodobacterales* as Drivers of *Ulva* Growth: From Macroalgal–Bacterial Interactions to Bioactive Factor Enrichment

**DOI:** 10.1007/s10886-026-01720-8

**Published:** 2026-05-19

**Authors:** Johann F. Ulrich, Simon B. Redlich, Anne Mohr, John Vollmers, Jörn Petersen, Thomas Wichard

**Affiliations:** 1 Institute for Inorganic and Analytical Chemistry, Friedrich Schiller University Jena, Lessingstr. 8, Jena, D-07743 Germany; 2 Institute for Biological Interfaces 5, Karlsruhe Institute of Technology, Karlsruhe, Germany; 3 Leibniz Institute DSMZ – German Collection of Microorganisms and Cell Cultures, Braunschweig, Germany; 4 Institute of Microbiology, Technical University of Braunschweig, Braunschweig, Germany

**Keywords:** Axenic cultures, Chromatography, Macroalgae, *Maribacter*, Morphogens, Natural product isolation, Phytohormone, *Roseovarius*

## Abstract

Marine bacteria significantly influence the development and productivity of algal communities. The green seaweed *Ulva* (*Chlorophyta)* relies on bacteria that secrete algal growth and morphogenesis-promoting factors (AGMPF). In a reductionist model system of *Ulva compressa* (cultivar *U. mutabilis*), the diverse microbiome can be substituted by two key bacteria, *Roseovarius* sp. MS2 and *Maribacter* sp. MS6, both of which release AGMPFs, thereby inducing cell division and cell differentiation. Our study examined various algal growth-promoting substances produced by marine *Rhodobacterales* (*Alphaproteobacteria*). The exploration survey indicated that 74 of the 97 examined strains demonstrated growth-promoting, cytokine-like action. These findings underscore the extensive impact of marine *Rhodobacterales* on *Ulva* growth. Bioactive fractions containing AGMPF activity were enriched from the culture supernatant of *Roseovarius* sp. strain MS2. Bioactive raw extracts were obtained through bioassay-guided fractionation and semi-preparative high-performance liquid chromatography; cell division-promoting properties were observed, but did not reach the activity level of the harvested supernatant. This study provides new insights that underscore the importance of bacterial–macroalgal interactions for marine ecosystems. Replacing bacteria with AGMPFs, including thallusin, enables the complete thallus formation of axenic *Ulva* cultures, facilitating various applications in biotechnology and aquaculture.

**Supplementary Information:**

The online version contains supplementary material available at 10.1007/s10886-026-01720-8.

## Introduction

Seaweeds provide essential ecological and economic benefits in marine environments; however, their performance depends on interactions with associated microbial communities, forming the seaweed holobiont (Saha et al. 2024; Wahl et al. 2012; Zilber-Rosenberg and Rosenberg 2008). Despite their importance, the molecular mechanisms and evolutionary processes underlying seaweed–microbe interactions remain poorly understood. Key challenges include identifying relevant microbial partners, understanding their recruitment, determining their functions, and assessing their roles across algal life stages, particularly under environmental stress (Ghaderiardakani et al. 2020).

**Fig. 1:**
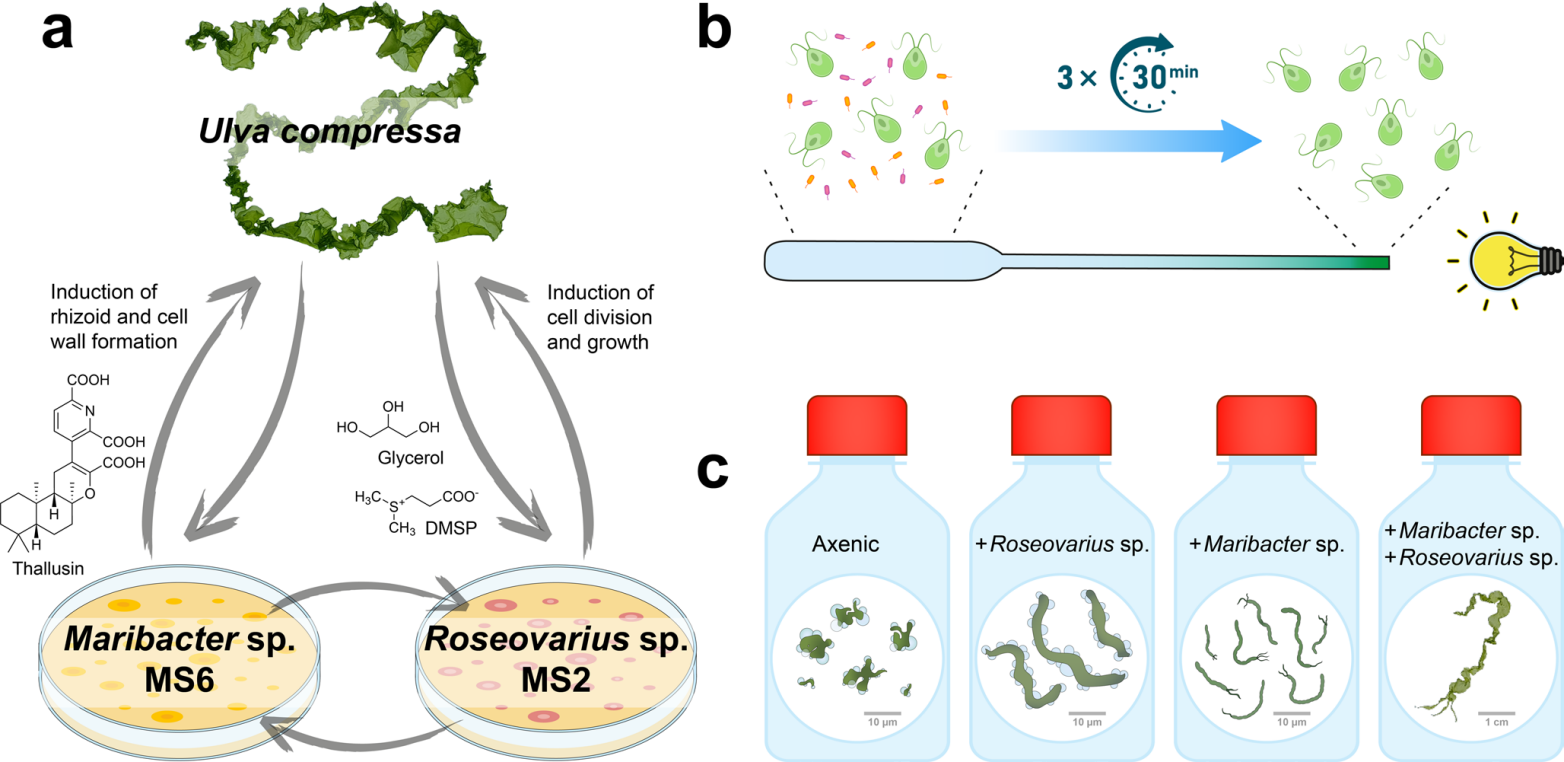
Overview of the symbiotic tripartite community between *Ulva compressa* and its associated bacteria, along with the experimental setup and morphotype categories. (**a**) Symbiotic interactions between *Ulva compressa*, *Maribacter* sp. MS6 and *Roseovarius* sp. MS2. *Maribacter* sp. provides thallusin to *Ulva*, inducing rhizoid and cell wall formation. The chemotactic *Roseovarius* bacteria are attracted by *Ulva* via dimethylsulfoniopropionate (DMSP) and utilize glycerol as a carbon source (Kessler et al. 2018). *Roseovarius* produces unknown chemical factors that induce cell differentiation in *Ulva* (Alsufyani et al. 2020). Additionally, chemical interactions between the bacteria have been suggested but remain unexplored. (**b**) Purification of *Ulva* gametes. The xenic bacterial suspension was injected into a glass Pasteur pipette containing sterile UCM. The phototactic *Ulva* gametes moved towards a light source positioned at the opposite end of the capillary, leaving the bacteria on the injection side. After 30 min, the accumulated gametes near the light source were harvested. Following three repetitions, the gametes became axenic, which was verified by PCR. These axenic gametes were then used for morphogenesis-guided bioactivity assays. (**c**) Morphotype categories following the addition of *Ulva*-associated bacteria (see Method section and Fig. S1)

Algal growth- and morphogenesis-promoting factors (AGMPFs) are central regulators of macroalgal development (Alsufyani et al. 2020; Ghaderiardakani et al. 2019; Wichard 2023). These include nutrients, phytohormones, and bacterial signaling molecules such as thallusin, which are essential for *Ulva* (Chlorophyta) morphogenesis (Fig. 1). Marine microorganisms are a rich source of bioactive compounds with ecological and biotechnological relevance (Ibrahim and El-Sheekh 2023; Karthikeyan et al. 2022; Rotter et al. 2021). In particular, members of the *Roseobacteraceae* and *Flavobacteriaceae* are known to produce growth-inducing factors (Grueneberg et al. 2016; Matsuo et al. 2005; Singh and Reddy 2014; Spoerner et al. 2012; Wichard 2015). Given the ecological and economic importance of macroalgae (Bolton et al. 2016; Dominguez and Loret 2019; Zhang et al. 2019), understanding AGMPFs, many of which cannot be synthesized by the algae themselves, is essential (Wichard 2023).

*Ulva compressa* (cultivar *Ulva mutabilis* Føyn) has emerged as a model system for studying bacteria–macroalgae interactions (Blomme et al. 2023). Two key symbiotic bacteria, *Maribacter* sp. MS6 (Flavobacteriaceae, Bacteroidota) and *Roseovarius* sp. MS2 (Roseobacteraceae, Pseudomonadota), form a tripartite community essential for normal *Ulva* development (Spoerner et al. 2012). In their absence, *Ulva* exhibits an undifferentiated callus-like morphotype. *Maribacter* sp. promotes rhizoid and cell wall formation via the AGMPF (−)-thallusin (Alsufyani et al. 2020), a highly potent morphogen (half maximal effective concentration: EC_50_ = 4.9 pmol/L) with hormone-like activity that can substitute for the bacterium (Dhiman et al. 2022). In contrast, the AGMPFs produced by *Roseovarius* sp. exhibit cytokinin-like properties but remain chemically unresolved (Alsufyani et al. 2020; Spoerner et al. 2012).

Phytohormones are low-molecular-weight compounds active at very low concentrations within the algal chemosphere, where organisms interact via signaling molecules, nutrients, and defense compounds (Alsufyani et al. 2017). These compounds regulate growth and stress responses in both terrestrial plants and algae (Ciura and Kruk 2018; Tarakhovskaya et al. 2007). Bacteria can also produce phytohormones, influencing host development by releasing exogenous signals (Costacurta and Vanderleyden 1995; Kurepin et al. 2014; Poveda and González-Andrés 2021; Spaepen 2015).

While thallusin from *Maribacter* spp. has been extensively studied (Alsufyani et al. 2017; Matsuo et al. 2005; Weiss et al. 2017; Wienecke et al. 2024), the compounds released by *Roseovarius* sp. MS2 remain unknown. We therefore aimed to investigate the distribution of AGMPF activity among *Alphaproteobacteria* of the order *Rhodobacterales*. Based on previous work (Spoerner et al. 2012), we hypothesized that *Rhodobacterales* produce a mixture of morphogenetic factors rather than a single compound such as thallusin.

To address this, we combined large-scale screening with bioassay-guided fractionation in a three-step approach:

(i) assessing the prevalence of AGMPF activity across *Rhodobacterales* strains from the German Collection of Microorganisms and Cell Cultures collection (DSMZ);
(ii) developing a protocol for partial purification of bioactive fractions containing AGMPF activity from *Roseovarius* sp. MS2; and.
(iii) evaluating the activity of these fractions in combination with *Maribacter* sp. MS6. This workflow provides a foundation for future structural elucidation of AGMPFs.

By advancing our understanding of bacterial–algal interactions, this study also supports the development of biotechnological applications. In particular, the combination of AGMPFs enables the cultivation of fully axenic and morphologically complete *Ulva* cultures, which are valuable for controlled research systems and applications in aquaculture, cosmetics, and pharmaceuticals (Halla et al. 2018; McKenna et al. 2025; Wichard et al. 2026).

## Methods and Materials

### Biological Material and Cultivation Conditions

Haploid gametophytes of the cultivar *Ulva mutabilis* Føyn (sl-G[mt+]; morphotype ‘slender’; locus typicus: Ria Formosa, Portugal, strain FSU-UM5-1) were utilized for all bioassays. *U*. *mutabilis* (reclassified in *Ulva compressa* by Steinhagen et al. 2019) was grown in *Ulva* Culture Medium (UCM) under standardized laboratory conditions, with a light/dark cycle of 17/7 h and a light intensity of 40–80 mol photons m^2^ s^− 1^ at 18 °C ± 2°C (Califano and Wichard 2018; Wichard and Oertel 2010). *Maribacter* sp. strain MS6 (GenBank EU359911) and *Roseovarius* sp. strain MS2 (GenBank EU359909) were grown at 20 °C ± 2°C in a marine broth enriched UCM (50%, *v*/*v*; Roth, Karlsruhe, Germany) on an orbital shaker. For the *Rhodobacterales* survey, a set of 96 mesophilic, well-growing, genome-sequenced, and phylogenetically diverse marine strains (Tomasch et al. 2025) were provided by the Leibniz Institute DSMZ-German Collection of Microorganisms and Cell Cultures GmbH (DSMZ; Table S1). *Roseovarius* sp. MS2 was initially isolated from the algal cultivar (Spoerner et al. 2012), and it is closely related to *Roseovarius mucosus* (Grueneberg et al. 2016). The complete genome of *Roseovarius* sp. MS6 was established using long-read PacBio sequencing (available at the National Center for Biotechnology Information; Genbank: JBKHVF000000000.1; Assembly: GCA_051447875.1; BioProject: PRJNA828511). The Type (Strain) Genome Server was used for digital DNA-DNA hybridization and the assessment of taxonomic novelty (Meier-Kolthoff and Göker 2019).

### Study Design

We first screened 96 marine strains of the alphaproteobacterial order *Rhodobacterales* to identify those capable of phenocopying *Roseovarius* sp. MS2 in the tripartite community of *U. compressa* (Fig. 1a). Strains were selected according to their phylogenetic diversity and categorized according to their ability to induce morphogenesis, with *Roseovarius* sp. MS2 serving as a key reference. Culture enhancement focused on optimizing minimal media conditions for efficient AGMPFs isolation. Axenic *Ulva* cultures were initiated at the gamete stage with bacterial culture supernatants to evaluate morphogenetic effects during germination and propagule development. Subsequently, solid-phase extraction protocols were developed to isolate and purify active substances, followed by chromatographic separation.

### Preparation of Axenic Gametes of *Ulva compressa*

Chopping the thallus and removing sporulation inhibitors from the algal fragments initiated mature *U*. *compressa* gametogenesis (Vesty et al. 2015). The swarming inhibitor was removed through an additional medium exchange, and gametes were released from the gametangia on the third day after gametogenesis induction. The released gametes were purified from bacteria using sterile equipment in a laminar flow cabinet, taking advantage of the positive phototaxis of the gametes as described by (Califano and Wichard 2018) (Fig. 1b). Gametes were quantified via flow cytometry (Nahor et al. 2021). Axenic gametes were assessed using an *Ulva* bioassay array (Sarstedt, Nümbrecht, Germany) in 24-well microplates, with each well filled with 1 mL UCM and inoculated with 50 to 100 gametes (Grueneberg et al. 2016).

### *Ulva* Bioassay Array to Determine the Morphogenetic Activity of Bacteria

Purified gametes were inoculated with *Maribacter* sp. MS6 and the corresponding bacteria of the *Roseobacter* clade (final concentration: OD_620_ = 1 × 10^− 4^) in 200 µl UCM using 96-well microplates. Triplicates of the microplates were achieved using a replicator with 96 pins (CR1000, Enzyscreen BV, Netherlands; (Grueneberg et al. 2016). Controls were included to compare the phenocopy of the reference strains (Fig. 1c). The inoculated cultures were kept in the dark for 24 h to allow the gametes to settle to the bottom of the culture tissue flask. After 7 and 14 days of growth under standard culture conditions, germling development was examined with an inverted Leica DMIL LED microscope (Leica, Solms, Germany) equipped with a digital camera (Nikon, Düsseldorf, Germany). All experimental procedures were conducted under strictly sterile conditions in a laminar flow cabinet (Califano and Wichard 2018).

Representative morphogenetic phenotypes of *U. compressa* after 2 weeks of cultivation were classified into four categories according to Grueneberg et al. (2016) (Figs. 1, S1a-d):

1. Axenic morphotype: Undifferentiated growth with irregular cell aggregates and pronounced cell wall protrusions.
2. *Roseovarius*-type morphotype: Thallus growth occurs; however, cell walls are irregular and damaged with protrusions, no rhizoid formation.
3. *Maribacter*/thallusin-type morphotype: Formation of rhizoids and intact cell walls and rhizoid formation, but a thallus is not formed.
4. Tripartite morphotype (*Ulva*–*Roseovarius*–*Maribacter*): Complete morphogenesis, including proper thallus development with rhizoid formation, and intact cell walls.

The survey screened in particular for those strains which phenocopied the *Roseovarius*-type and complemented the activity *Maribacter* sp.

### Phylogenetic Analysis of *Rhodobacterales*

To explore the phylogenetic relationships among the 97 genome-sequenced *Rhodobacterales* strains investigated in this study (Table S1), a phylogenomic Neighbor-joining tree (NJ) was calculated as recently reported (Tomasch et al. 2025). In short, Proteinortho5 (Lechner et al. 2011) analyses of the genomes deposited at the NCBI database resulted in the detection of 633 single-copy orthologs shared across all comparison strains. Orthologeous sequences were aligned using MUSCLE software (Edgar 2004). To compare only fully alignable protein regions, any trailing C- and N-terminal overhangs were clipped, and internal hypervariable regions were subsequently filtered using Gblocks, 2000), resulting in a final alignment of 176,470 conserved amino acid positions. A neighbor-joining tree was constructed using MEGA X software, applying the Tamura–Nei model for evolutionary distance estimation. The robustness of the tree topology was evaluated through 1,000 bootstrap replicates, with only bootstrap values above 50% shown in the final tree.

The resulting phylogenetic tree was color-coded to indicate the functional phenotypes of each strain regarding their ability to induce morphogenesis in *Ulva*. Specifically, strains including the MS2-like morphotype were marked in green, those associated with the axenic morphotype in red, and those producing previously uncharacterized or “new” morphotypes in blue. This functional annotation provided insight into the phylogenetic distribution of morphogenetic traits within the different clades of marine *Rhodobacterales*.

### Preparation and Harvesting of the *Roseovarius* sp. MS2 Supernatant

*Roseovarius* sp. MS2 was cultivated in a specific minimal medium comprising UCM with 1% glycerol (*v*/*v*). For this purpose, 500 µL of a stationary *Roseovarius* culture comprising 50% marine broth (Roth, Germany) and UCM (*v*/*v*) was inoculated with 500 mL UCM containing 1% glycerol (*v*/*v*). After 1 week, the culture was harvested at an optical density of OD_620_ = 0.1. The culture was centrifuged (9,000 rpm, 20 min, 10 °C) and sterile-filtered twice (PES Membrane Filter, 0.22 μm pore size, Merck, Germany). The sterile-filtered culture supernatant was frozen and stored at − 20 °C prior to use. Dilutions of the supernatant in UCM (1%, 5%, 10%, 25%, 50%, 75%, and 100% supernatant (*v*/*v*)) were prepared for the *Ulva* bioassay array. Bioassays were performed in 24-well microplates using the *Ulva* bioassay array as described above, with 1 mL of each dilution replacing the bacterial strain at pH 8.0–8.2.

### Evaluating the Heat and UV Stability of the *Roseovarius* sp. MS2 Supernatant Bioactivity

For the heat stability assay, 50 mL aliquots of the sterile-filtered *Roseovarius* supernatant (harvested at OD_620_ = 0.1) were subjected to three different conditions: one set was placed in a water bath and boiled at 100 °C for 1 h, another set was maintained at room temperature (20 °C) as a control, and a third set was frozen at − 25 °C to assess the storability at low-temperature.

For the UV/vis stability assays, 50 mL aliquots of the supernatant were exposed to ultraviolet light for 4 h. Two different wavelengths were tested: UV-C (254 nm) and UV-A (366 nm). A dark control was prepared by covering the supernatant with aluminum foil to prevent light exposure. All UV treatments were conducted in sterile 50-mL centrifuge tubes (Sarstedt, Germany) positioned 10 cm underneath the light source. After treatment, all samples were maintained in sterile conditions at room temperature until further bioassay testing.

Morphogenesis-guided *Ulva* bioassays were conducted to assess the bioactivity of the treated supernatants. Axenic gametes were incubated in 24-well plates containing sterile UCM with 75% (*v*/*v*) of the treated supernatant. The bioassays were incubated for 14 days and subsequently analyzed by comparing the morphogenetic changes induced by the treated supernatants with those of the untreated controls to determine whether the bioactivity of the *Roseovarius* supernatant was affected by heat or UV exposure.

### Solid-Phase Extraction of *Roseovarius* Supernatant

To characterize and optimize the extraction of the bioactive *Roseovarius* factors, four solid-phase extraction (SPE) cartridges with different sorbent properties were tested: Oasis^®^ HLB Plus (Waters Corporation, USA) (hydrophilic-lipophilic balanced copolymer), Chromabond^®^ EASY (Macherey-Nagel, Germany) (polystyrene-divinylbenzene copolymer), Chromabond^®^ HILIC (Macherey-Nagel, Germany) (zwitterionic silica-based phase), and HyperSep™ Hypercarb™ (Thermo Fisher Scientific, USA) (porous graphitic carbon). These cartridges were selected to assess the polarity and charge properties of the compounds. The Oasis HLB and Chromabond EASY cartridges operate via reversed-phase interactions, whereas the Chromabond HILIC cartridge is optimized for the retention of polar and ionic compounds. The HyperSep™ Hypercarb cartridge was used to evaluate the effect of pH on adsorption efficiency, as the manufacturer suggests that retention is pH dependent.

Bacterial cultures of *Roseovarius* sp. MS2 were cultured until optical densities (ODs) of approximately 0.1, 0.6, and 1.2 were reached. At each OD stage, replicate cultures (*n* = 3) were harvested and centrifuged at 9,500 rpm for 20 min at 10 °C. The supernatants (3 × 250 mL) were decanted, sterile-filtered, pooled, and aliquoted into 45 mL portions before storage at − 20 °C. A 10 mL reference sample was retained from each batch of supernatant.

Each SPE cartridge type was tested in replicates (*n* = 3), with 45 mL of *Roseovarius* supernatant loaded per cartridge. For the HyperSep™ Hypercarb™ cartridge, the effect of pH on retention was examined by adjusting the pH of 60 mL supernatant to 6, 7, and 8 using 1 M NaOH or 1% HCl (*v*/*v*). Each pH-adjusted supernatant was divided into 20 mL aliquots and processed accordingly.

Flow-through and wash fractions were collected during extraction. Eluates, wash solutions, and 10 mL of flow-through from the Chromabond HILIC cartridge (collected after 20–30 mL had passed through) were evaporated to dryness under nitrogen at 20 °C. Dried samples were reconstituted in 0.5 mL of UCM, whereas HILIC flow-through fractions were reconstituted in 2 mL of UCM. All sterile-filtered extracts, wash solutions, and flow-through fractions were subsequently prepared for bioactivity assays by mixing 100 µL of extract with 100 µL of gamete-UCM suspension.

### Purification and Bioassay-guided Fractionation for the Enrichment of AGMPFs

For purification, 500 mL of centrifuged and sterile-filtered bacterial culture supernatant was solid-phase extracted using an Oasis HLB Plus cartridge. The cartridge was preconditioned with 5 mL of methanol and equilibrated with 10 mL of MicroPure water (TKA Wasseraufbereitungssysteme GmbH, Germany). The culture supernatant was then loaded onto the cartridge. The HLB matrix was washed with 10 mL MicroPure water. The matrix was eluted first with 4 mL of 50% (*v*/*v*) MeOH and then with 100% MeOH. Biological activity was observed only in the 50% (*v*/*v*) MeOH fraction. The eluate solvents were then evaporated with nitrogen (Biotage-TurboVap LV, Uppsala, Sweden). The extracts were subsequently dissolved in 500 µL of 50% (*v*/*v*) MeOH and 100% MeOH, respectively. The solution was filtered (PVDF, 0.22 μm, 4 mm, Millex-GV), transferred to ultra-high performance liquid chromatography–mass spectrometry (UHPLC–MS) vials, and stored at − 20 °C until performing further purification steps and the *Ulva* bioassay array.

For bioassay-guided fractionation, semi-preparative UHPLC (UltiMate HPG-3400 RS binary pump) was performed coupled to an ESI-ISQ mass spectrometer (Thermo Fisher Scientific, Germany). The Gemini C-18 RP semi-preparative chromatography column (250 × 10 mm; 5 μm; 110 Å; Phenomenex, Aschaffenburg, Germany) was maintained at 20 °C. Eluent A comprised 2% (*v*/*v*) acetonitrile and 0.1% (*v*/*v*) formic acid in H_2_O, whereas pure acetonitrile was used as eluent B. The following conditions were applied: −3.0 min to 0.0 min (equilibration; 0% B), 0.0 min to 0.2 min (0% B), 0.2 min to 18.0 min (100% B), 18.0 min to 27.5 min (100% B), 27.5 min to 28.0 min (0% B), and 28.0 min to 35.0 min (0% B). All measurements were performed with a constant flow rate of 2.0 mL min^− 1^. Samples were injected using a WPS-3000 autosampler equipped with a 100 µL injection syringe and maintained at 10 °C.

For the *Ulva* bioassay array of the extracts, 2 µL of the extract obtained from the solid phase extraction was added to the 24-well microplates containing axenic gametes in 1 mL UCM, resulting in a MeOH ratio of 1:1000, which does not harm algal development. The dried semi-preparative purified fractions from HLB extract were dissolved in 100 µL of 50% MeOH (*v*/*v*); 2 µL of each fraction was tested in the 24-well plates with axenic gametes in 1 mL UCM.

### Instrumental Methods of Chemical Analysis

UV/vis spectra were recorded during chromatography with a Dionex UltiMate 3000 instrument at 290 nm. Mass spectra were recorded using electrospray ionization, and the ion single quadrupole (ISQ) mass spectrometer (Thermo Fisher Scientific) was coupled. To collect purified fractions, a post-column adjustable flow splitter (ASI QuickSplit 600-PO10-04, USA) was used, allowing 1/10 of the sample to be directed into the MS while 9/10 was collected. The fractions were manually collected in 4 mL glass vials and evaporated under nitrogen (Biotage-TurboVap LV, Uppsala, Sweden). The total ion current (TIC) was used in positive ionization mode. The following MS parameters were applied: mass range of 100–1000 *m*/*z*, vaporizer temperature of 450 °C, ion transfer tube temperature of 300 °C, source voltage for positive ions at 3.0 kV, sheath gas pressure at 4.8 bar, auxiliary gas pressure at 0.7 bar, and sweep gas pressure at 0.007 bar.

### Microscopic Analysis of the *Ulva* Bioassays

The bioassays of axenic gametes inoculated with raw extracts were analyzed after 14 days using an inverted microscope (Leica Microsystems CMS GmbH, Wetzlar, Germany). Images were captured with a microscope-compatible digital camera (Nikon Digital Sight DS-Fi2, DS-U3). The qualitative features observed were as follows: (i) the presence of vesicular cell wall protrusions, (ii) the absence of rhizoids (both in the absence of the *Maribacter* sp. MS6 factor thallusin), and (iii) longitudinal growth in the presence of the *Roseovarius* sp. MS2 factor (Spoerner et al. 2012). The presence of the *Roseovarius* sp. MS2 factor, associated with significant growth in size and length, was quantified using ImageJ (Schneider et al. 2012).

### Quantitative Analysis of the Algal Surface Area

After microscopic examination, the acquired images were analyzed with ImageJ. The surface area of individual algae was measured as follows. The image color was first converted to grayscale (*Image* ◊ *Type* ◊ *8-bit*). Then, the scale was set (*Analyze* ◊ *Set Scale*) with 83 pixels corresponding to 100 μm. Background impurities were removed using ‘Remove Outliers’ (*Process* ◊ *Noise* ◊ *Remove Outliers*; Radius: 10.0 pixels; Threshold: 1; Which outliers: dark). The threshold for the algae image was generated using the automatic routine (*Process* ◊ *Binary* ◊ *Make Binary*), resulting in black algae on a white background. The algae surface areas were then determined (*Analyze* ◊ *Analyze Particles*; Size pixel²: 1,000-100,000; Circularity: 0.00–1.00; Show: Outlines; Check ‘Display Results’). All algae were within the selected range.

The determined values were checked for outliers using Dixon and Dean’s method (*p* < 0.05) (Reichenbächer and Einax 2011): For area determinations, the highest control value at 49,456 μm², the lowest value of the 1% supernatant + MS6 at 2,534 μm², and the highest value of the 75% supernatant at 40,264 μm² were removed as outliers. For length measurements, the highest value of the 0% supernatant at 560 μm, the highest control value at 1,399 μm, and the highest value of the 25% supernatant + MS6 at 1,034 μm were removed as outliers.

All data followed a normal distribution (Kolmogorov–Smirnov test, *p* > 0.05, OriginPro 2024 Software, USA). Homogeneity of variance was confirmed using the Levene test (*p* < 0.05, OriginPro 2024 Software, USA). For datasets with homogeneous variances, an ANOVA with Tukey’s post-hoc test (*p* < 0.05) was performed; otherwise, a Welch-ANOVA (*p* < 0.05) was used to determine significance.

## Results

### Prevalence and Abundance of AGMPFs Among Marine *Rhodobacterales*

**Fig. 2:**
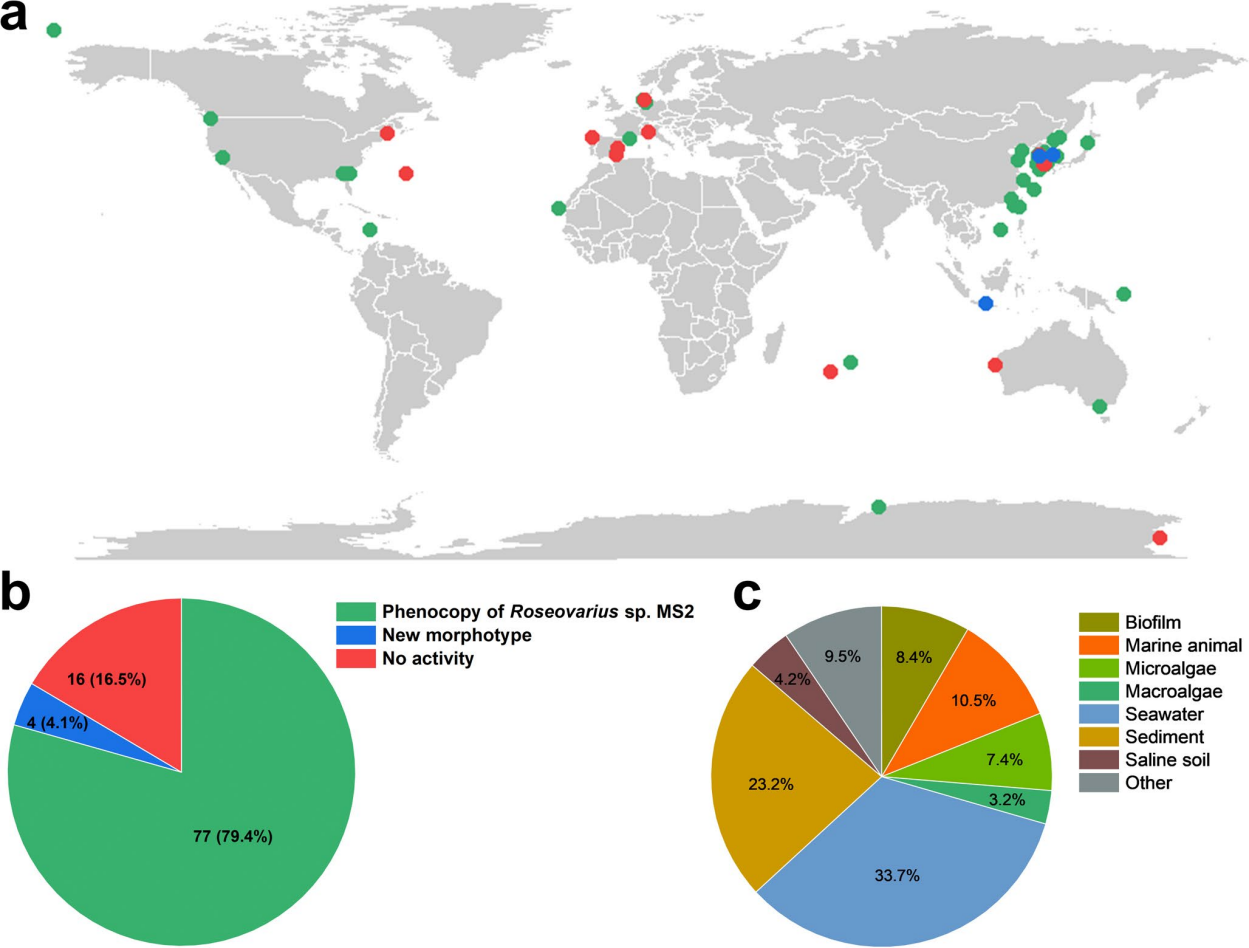
Morphogenetic activity test of 97 marine *Rhodobacterales* strains by cocultivation with sterile *Ulva compressa* gametes (**a**, **b**) World map and pie chart showing the isolation sites and proportion of strains inducing the characteristic *Roseovarius* sp. MS2-like morphotype (green, 79.4%), non-inducing strains (red, 16.5%), and strains causing a “new” morphotype (blue, 4.1%). (**c**) Pie chart of isolation sources: 29.5% from biofilms, algae, or marine animals; 33.7% from seawater; and 36.9% from sediments, saline soil, or other sources (see also Table S1)

We screened 96 strains of bacteria from marine *Rhodobacterales* (synonym: Roseobacter clade) collected worldwide and curated by the DMSZ to identify those capable of phenocopying *Roseovarius* sp. MS2 in the tripartite model community (*Ulva compressa*–*Maribacter* sp. MS6–*Roseovarius* sp. MS2) using a morphogenetic activity bioassay (Fig. 2a). We found that 79.4% of these strains, spanning the entire evolutionary depth of *Rhodobacterales*, successfully substituted *Roseovarius* sp. MS2 in the tripartite community (Fig. 2b) and promoted cell division and longitudinal growth; however, these strains did not induce the rhizoidal zone and normal cell wall formation . Conversely, 16.5% failed to replace *Roseovarius* sp. and displayed the axenic morphotype. Bacteria were further classified according to their origin as stated in the Bacterial Diversity Metadatabase (BacDive; (Schober et al. 2024) of the DSMZ culture collection (Fig. 2c).

Growth of *U. compressa* was complemented by 77 of the 96 strains tested. As these strains are distributed across all twelve described clades of the *Rhodobacterales* as well as the genus *Litoreibacter*, this demonstrates that the factor produced by *Roseovarius* sp. MS2 to induce cell divisions (Fig. 1a) is widely conserved among marine *Rhodobacterales*.

The bacterial isolates exhibited a broad global distribution, with most originating from tropical and subtropical regions and some from temperate and polar zones (Fig. 2a). Bacterial isolates originated from various environments, including open saltwater, coastal areas, surface and deep-sea waters, sediments, and associations with algae, animals, or saline soils (Fig. 2d). The distribution of isolation sources was approximately equally divided among seawater, sediment, and biological sources. Inducing strains were widely distributed across these habitats, with no significant difference in induction activity across strains associated with flora (80% of flora-associated strains, 8 out of 10) and those isolated from marine fauna (70% of fauna-associated strains, 7 out of 10). Among the flora-associated strains, only three were isolated from macroalgae, all of which were inducing strains; the remaining 7 flora-associated strains were isolated from microalgae (diatoms and dinoflagellates). Most non-inducing strains originated from seawater, marine fauna, microalgae, or biofilms. Overall, no direct correlation was found between morphotype induction and the isolation source or geographic origin.

A limited number of the samples originated from the deep sea (below 200 m). *Celeribacter indicus* was isolated from a depth of 2,946 m and exhibited no inducing activity. *Pseudophaeobacter arcticus* was obtained from a depth of 167 m in the Arctic Ocean, near the deep-sea boundary. Notably, an inducing strain, *Pseudooceanicola nanhaiensis*, was isolated from deep-sea sediment at a depth of approximately 1,500 m.

Microscopic examination revealed that most inducing strains accumulated around the rhizoidal zone of *U. compressa*, indicating biofilm formation, similar to the reference strain *Roseovarius* sp. MS2, which served as a positive control in the *Ulva* bioassay (Fig. S1e, f). However, 12 inducing strains showed no rhizoidal accumulation, indicating the presence of diverse mechanisms. To further characterize the AGMPFs, we selected *Roseovarius* sp. MS2 from the tripartite model system after optimizing culture conditions and establishing a methodology for solid-phase AGMPF extraction.

### Phylogenetic Analysis of the Tested *Rhodobacterales* Strains and Their Morphogenic Activity

**Fig. 3:**
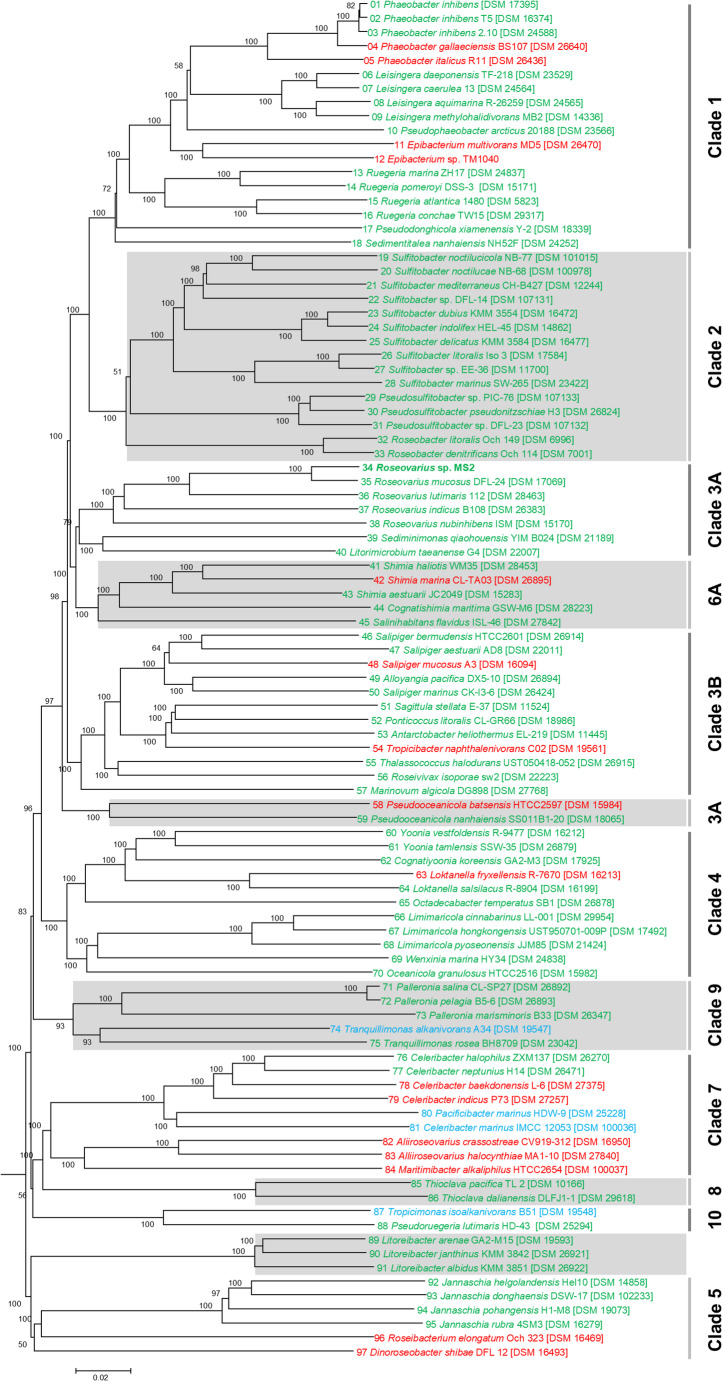
Phylogenomic neighbor-joining tree of 97 *Rhodobacterales* strains investigated in this study. The protein tree was calculated from an alignment of 176,470 amino acid positions across 633 orthologous genes. The phylogenetically broad taxon sampling includes marine strains from twelve different clades of the *Roseobacter* group (Table S1). The tree illustrates the induction of *Ulva* morphotypes compared to the reference strain *Roseovarius* sp. MS2: Phenocopy of *Roseovarius *sp. MS2 (green), axenic (red), and “new” (blue) morphotype. Clades with exclusively inducing strains (e.g., Clades 2 = 100%) indicate conserved morphogenetic activity, whereas clades containing both inducing and non-inducing strains (e.g., Clade 1 = 78%, Clade 3B = 83%, Clade 4 = 91% or Clade 7 = 44% inducing strains, for *n* ≥ 9) suggest more functional divergence within closely related taxa

The phylogenomic analysis revealed twelve distinct and well-supported clades within the order *Rhodobacterales*, which is in agreement with a recent comprehensive analysis of more than 300 genomes of this order (Tomasch et al. 2025), thus confirming the expected evolutionary relationships among strains investigated in this study. Major genera, including *Phaeobacter*, *Leisingera*, *Ruegeria*, and *Sulfitobacter*, formed coherent subtrees with high bootstrap support (typically ≥ 98%), confirming the established taxonomy and providing a robust framework for functional interpretation (Fig. 3).

Importantly, *Roseovarius* sp. MS2, the key strain in the *Ulva* tripartite community model, was found to occupy a well-defined position in clade 3 A of the phylogenetic tree, closely related to *Roseovarius mucosus* DSM 17,069^T^ (Fig. 3). A TYGS analysis (i) confirmed that *R. mucosus* is the closest related type strain and (ii) resulted in a digital DNA-DNA hybridization value of 33.0% (formula *d*_*4*_), providing clear evidence that *Roseovarius* sp. MS2 represents a potential new species (threshold: 70%). All six investigated strains of clade 3 A, representing the genera *Roseovarius*, *Sediminimonas*, and *Litorimicrobium*, induce MS2-like morphogenesis in the *Ulva* assay. The phylogenomic analysis confirmed the common branching of clade 3 A and clade 6 A (Tomasch et al. 2025), which comprises strains of the genera *Shimia*, *Cognatishimia* and *Salinihabitans*, and proposed a well-supported sister group relationship with clade 1 (*Phaeobacter*, *Leisingera*, *Ruegeria*) and clade 2 (*Sulfitobacter*, *Pseudosulfitobacter*, *Roseobacter*; Fig. 3).

The inclusion of morphotype-induction phenotypes, color-coded within the phylogenetic tree, further emphasized that most *Rhodobacterales* strains could phenocopy *Roseovarius* sp. MS2 (77/96; Fig. 2b); however, interestingly, non-active strains were scattered “randomly” except one interesting group comprising *Aliiroseovarius crassostreae*, *Aliiroseovarius halocynthiae*, and *Maritimibacter alkaliphilus* (Fig. 3). Some notable features were observed during the bioassays for four strains (4% of the total tested). *Celeribacter marinus* and *Pacificibacter marinus*, both isolated from seawater in South Korea, induced a previously unobserved *Ulva* morphotype (Fig. S2). However, although propagules initially developed, they later exhibited blade cell darkening and death, suggesting a potential algicidal effect. Noteworthy *Tropicimonas isoalkanivorans*, isolated from Indonesian seawater, induced a delayed but partial recovery of algal germlings showing irregular thallus formation. In addition, *Tranquillimonas alkanivorans* triggered a distinctive branching pattern in the thallus (Fig. S2). All those morphotypes different from the typical phenocopy of *Roseovarius* sp. MS2 warrants further investigation.

### Characterization of the Bioactivity and Stability of the *Roseovarius* Factor in *Ulva* Growth Medium

**Fig. 4:**
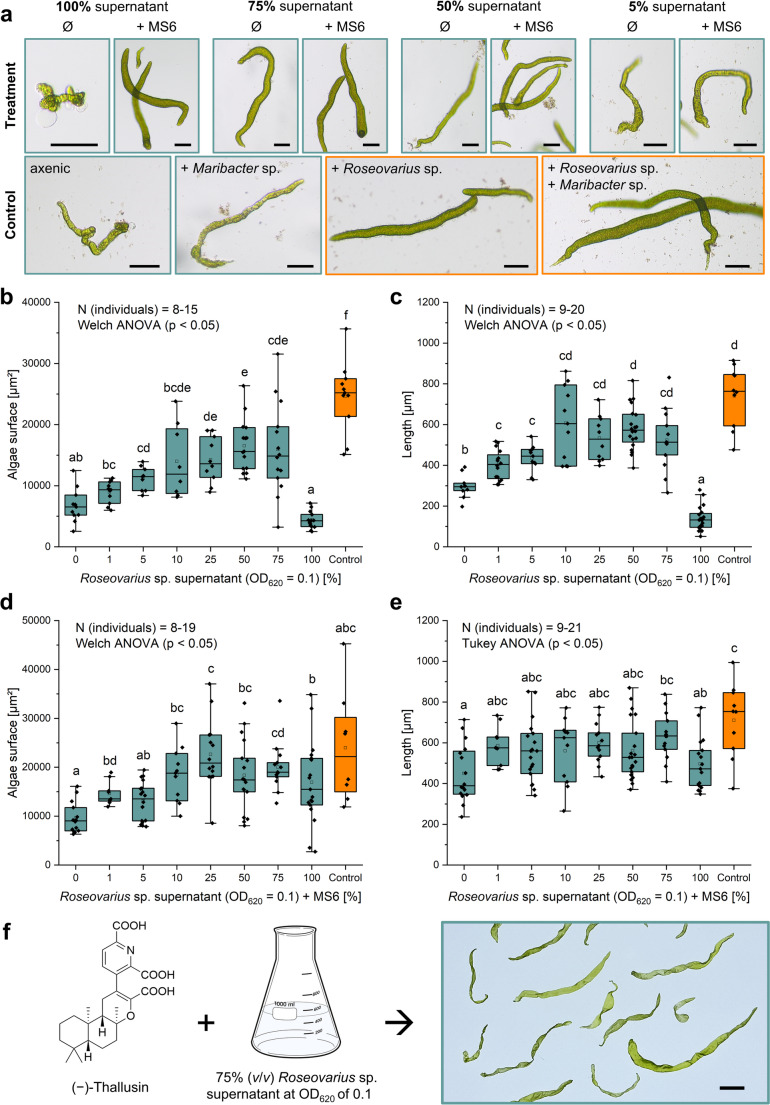
Quantification of *Roseovarius* supernatant activity to establish a protocol for the cultivation of fully developed axenic *Ulva* considering surface and longitudinal growth. (**a**) *Ulva* bioassays showing the effect of varying relative volumes (*v*/*v*) of double sterile-filtered *Roseovarius* culture supernatant, without (Ø) and with complementation by *Maribacter* sp. MS6. Controls are displayed in the lower section: the *Roseovarius* sp. MS2 control is outlined in orange, and the tripartite community control (*Roseovarius* sp. MS2 + *Maribacter* sp. MS6) in yellow. Cultures were evaluated after 14 days. Scale bar = 100 μm. (**b**, **c**) Surface (b) and longitudinal (c) growth of *Ulva compressa* in response to different proportions of *Roseovarius* supernatant. The positive control (orange) represents the bipartite *Ulva–Roseovarius* sp. MS2 community. (**d**, **e**) Surface (d) and longitudinal (e) growth of *U. compressa* with varying proportions of *Roseovarius* supernatant, supplemented with *Maribacter* sp. MS6. The positive control (orange) represents the tripartite *Ulva–Roseovarius–Maribacter* community. In panels (b–e), different letters indicate significant differences in algal growth (surface area or length) between treatments (Welch’s ANOVA for non-homogeneous variance and one-way ANOVA with Tukey’s post hoc test for homogeneous variance, *p* < 0.05, *n* = 8–21). Box-and-whisker plots represent the 90th/10th percentiles (whiskers), 75th/25th percentiles (boxes), and the median (center line). (**f**) Schematic of the cultivation protocol for fully developed axenic *Ulva* spp. cultures. Axenic *Ulva* gametes were incubated in a medium containing 75% (*v*/*v*) sterile-filtered *Roseovarius* culture supernatant and 25% *Ulva* Culture Medium (UCM), supplemented with (−)-thallusin (2 × 10⁻⁸ mol L⁻¹). After 6 weeks of incubation, fully developed axenic cultures were harvested. Scale bar = 1.0 cm

Bioactivity assays were microscopically evaluated after two weeks, focusing on algal longitudinal (length) and size (surface area) growth (Fig. 4a). Both parameters exhibited identical trends (*compare* Fig. 4b *with c*), demonstrating that the *Roseovarius* factor proportionally stimulated growth in both dimensions; thus, these metrics were reliable for quantification. The assays showed that increasing proportions (*v*/*v*) of the AGMPF-containing supernatant significantly enhanced *Ulva* growth (Welch ANOVA, *p* < 0.05). Longitudinal and surface growth increased in a dose-dependent manner, reaching a plateau at ~ 10% (*v*/*v*), with an EC_50_ of 5.5% (*v*/*v*) (Fig. S3). Higher concentrations (50–75%) supported sustained growth, potentially due to uptake of the *Roseovarius*-derived factor by *Ulva* (Fig. 4b, c).

Interestingly, the pure supernatant (100%) supported *Ulva*’s growth only in the presence of *Maribacter* sp. MS6. In the absence of *Maribacter* sp., the supernatant exhibited toxicity to the algae under standard culture conditions (*compare* Fig. 4b with *d* and Fig. 4c with *e*), a phenomenon warranting further investigation.

To assess the physicochemical properties of the *Roseovarius* factor, a double-sterile-filtered supernatant was irradiated with UV-A (366 nm) and UV-C (254 nm) for 4 h, with foil-shielded samples as controls. Additional treatments included boiling at 100 °C for 1 h, storage at room temperature (20 °C), and freezing at − 25 °C. All treatments retained bioactivity, indicating that the active compound(s) are stable to heat and UV exposure (Fig. S4). Consequently, no special protective measures were required during subsequent purification at ambient temperature. However, as such harsh treatments may alter the surrounding matrix, they were not pursued further.

### Cultivation of Axenic *Ulva* Cultures By Bacterial Supernatant Supplement

**Fig. 5:**
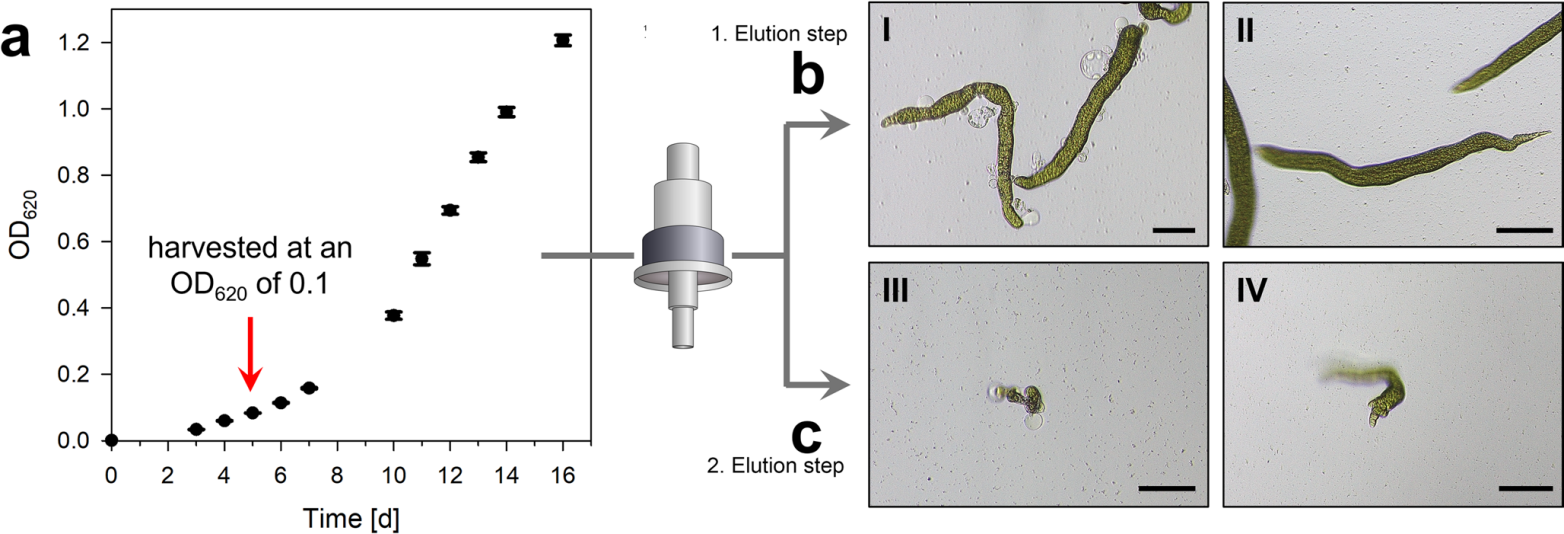
Workflow for isolating the *Roseovarius* derived factor from sterile-filtered supernatant using HLB solid-phase extraction (SPE) and two-step elution. (**a**) Growth curve of *Roseovarius* sp. MS2 in UCM supplemented with 1% glycerol (v/v) (*n* = 3). The culture was harvested at an optical density (OD₆₂₀) of 0.1 (indicated by the arrow), centrifuged, and then sterile-filtered through a 0.22 μm membrane. The supernatant was subjected to SPE, followed by sequential elution with (**b**) 50% methanol (MeOH, v/v) and (**c**) 100% MeOH. Morphogenesis-guided *Ulva* bioassays were evaluated microscopically after 2 weeks applying the MeOH extracts: (I) The 50% MeOH extract contained the *Roseovarius* factor . (II) The 50% MeOH extract supplemented with *Maribacter* sp. restored full morphogenesis . (III) The 100% MeOH extract and (IV) 100% MeOH extract supplemented with *Maribacter* sp. were inactive . Scale bar = 100 μm

To enable axenic growth of *Ulva*, a specific culture medium, termed *Axenic Ulva Growth Medium (AUGM)*, was established. AUGM comprises 75% (*v*/*v*) sterile-filtered *Roseovarius* supernatant and 25% UCM supplemented with synthetic (−)-thallusin at a final concentration of 2 × 10⁻⁸ mol L⁻¹. This medium effectively supported both the growth and morphogenesis of *Ulva* in the complete absence of associated bacteria and was renewed every 2 weeks under the standardized conditions. In experimental trials, *Ulva* gametes inoculated into AUGM reliably developed into healthy thalli, demonstrating the robustness and efficacy of this axenic culture system (Fig. 4f). Regarding the AUGM preparation, a key prerequisite for retaining the bioactivity of the *Roseovarius* supernatant is maintaining a low bacterial density. This condition prevents the accumulation of potentially harmful metabolites produced during the stationary growth phase and ensures optimal morphogenetic induction in *Ulva*. Therefore, we recommend cultivating *Roseovarius* in a minimal medium (UCM + 1% glycerol, *v*/*v*) until an OD₆₂₀ of 0.1 is reached, corresponding to the early exponential phase, after 5 days of incubation. The late exponential phase is reached after 16 days at OD_620_ = 1.2 (Fig. 5a). The culture should then be centrifuged, sterile-filtered to remove bacterial cells, and the supernatant stored at − 25 °C until use. Notably, the morphogenetic activity of the supernatant remained stable for at least 1 year under these storage conditions, supporting reproducibility and scalability in axenic *Ulva* cultivation.

### Extraction, Screening, and Partial Purification of AGMPFs from *Roseovarius* sp. Culture Medium

For AGMPFs isolation, the bacterial minimal medium (UCM + 1% glycerol, *v*/*v*) reduced the risk of contamination from complex media components and provided a clean matrix suitable for subsequent purification. Under these optimized conditions, *Roseovarius* was harvested at an optical density (OD₆₂₀) of 0.1.

The comparative evaluation of four cartridge types, Oasis HLB, Chromabond HILIC, Chromabond EASY and HyperSep Hypercarb, across different culture densities (OD₆₂₀ = 0.1, 0.3, 1.2) revealed clear differences in their ability to retain and recover bioactive compounds. Oasis HLB demonstrated the highest recovery efficiency, producing a strong eluate activity (+++, Table 1) at low culture density (OD₆₂₀ = 0.1). However, its performance declined markedly with increasing biomass, yielding only trace activity at higher ODs, suggesting capacity limitations or increased interference from matrix components.

**Table 1:** Bioassay results of *Roseovarius* sp. supernatants after solid-phase extraction (SPE) with different cartridges. Cultures were harvested at three growth phases (OD₆₂₀ = 0.1, 0.3, and 1.2), and the corresponding supernatants were subjected to SPE. The flow-through, wash fractions, and eluates were tested in the *Ulva* morphogenesis–guided bioassay. Activities were classified as follows: + (active), +++ (highly active), – (inactive), and t (toxic). Each assay was performed in triplicate (*n* = 3)

	OD_620_	Oasis HLB	Chromabond HILIC	Chromabond EASY	HyperSep Hypercarb
Activity of the throughput	0.1	**+**	**+**	**+++**	**+++**
	0.3	**+**	**+**	**+++**	**+++**
	1.2	**+**	**+**	**+**	**+++**
Activity of the wash throughput	0.1	**−**	**−**	**+**	**−**
	0.3	**−**	**−**	**−**	**−**
	1.2	**−**	**−**	**−**	**−**
Activity of the eluate	0.1	**+++**	**−**	**−**	t
	0.3	t	**−**	t	t
	1.2	t	**−**	t	t

Chromabond HILIC showed no detectable activity in the eluate under any condition, suggesting that it is unsuitable for isolating the target compounds. Chromabond EASY and Hypercarb application resulted in activity in the initial throughput, particularly at lower ODs, indicating weaker compound retention. Both yielded only trace levels of activity in the eluate at higher ODs. Hypercarb exhibited consistently low binding and recovery efficiency, with only trace activity observed in all eluates.

Across all cartridge types, recovery in the eluate tended to decrease as culture density increased, whereas activity losses in the throughput remained relatively stable or increased. This pattern suggests that higher biomass may cause saturation of binding sites, reduced compound accessibility, or enhanced losses during processing.

Overall, Oasis HLB was the most promising cartridge for recovering bioactive compounds from low-density cultures, whereas Chromabond^®^ HILIC and Hypercarb™ offered little benefit for this application. These results highlight the importance of both cartridge chemistry and culture density in optimizing recovery efficiency and should inform the design of future purification protocols for these bioactive factors (Table 1).

Using the Oasis HLB cartridge, crude extracts were prepared from the supernatant of *Roseovarius* sp. MS2 cultures. To simplify the eluate matrix and separate potential nonpolar toxic compounds from the targeted AGMPFs, a stepwise elution was performed, initially with 50% methanol–water (*v*/*v*) and followed by 100% methanol. The inducing compounds were efficiently recovered in the 50% methanol–water fraction (Fig. 5b), whereas the subsequent 100% methanol elution exhibited no detectable bioactivity (Fig. 5c), confirming the effectiveness and selectivity of the 50% methanol–water fraction for isolating the active compounds (Fig. 5b, c).

**Fig. 6:**
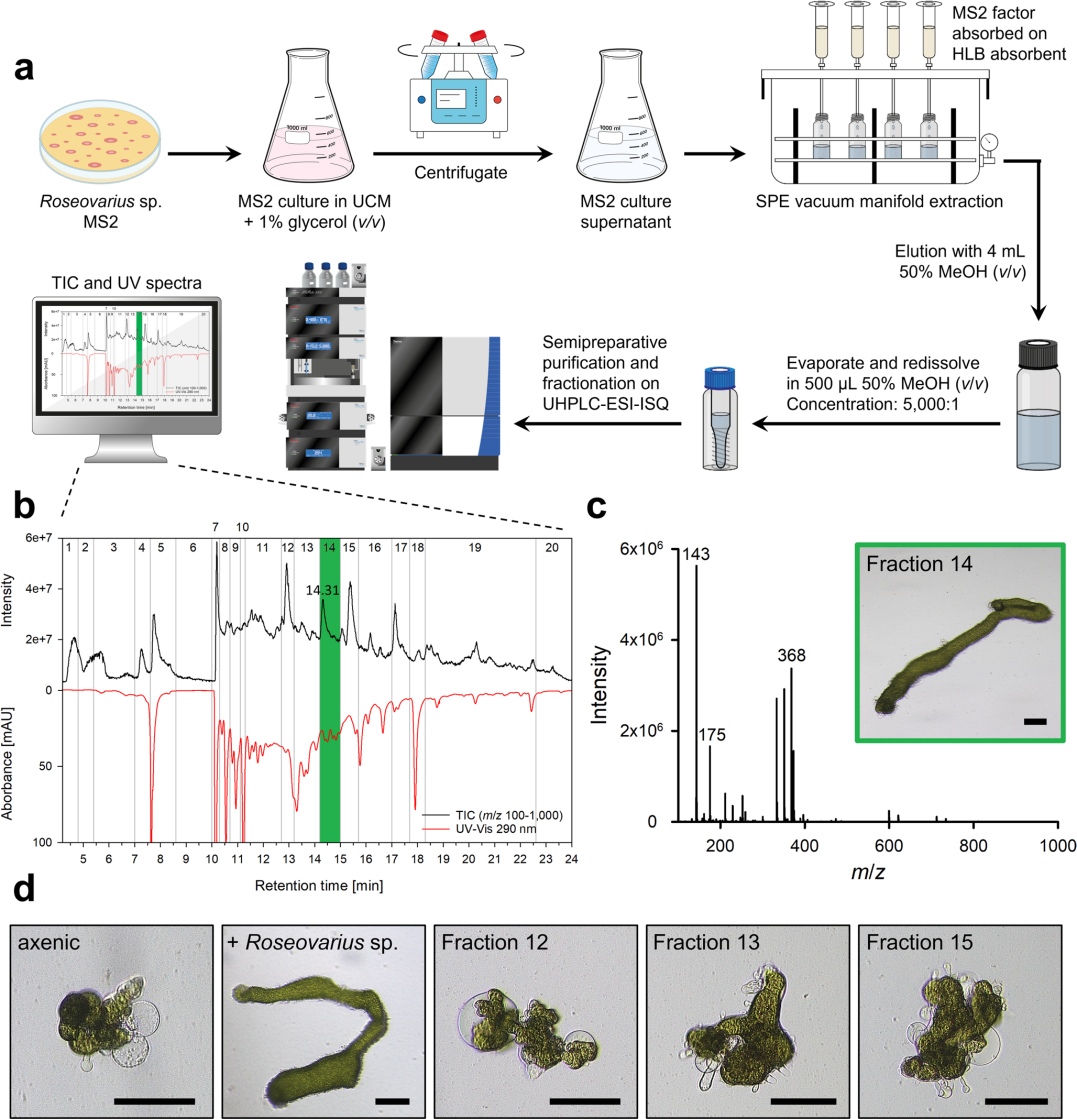
Scheme of partial purification and identification of growth-promoting activities in the supernatant of *Roseovarius* sp. MS2 (**a**) Step-by-step workflow starting with the cultivation of *Roseovarius* sp. in a minimal medium consisting of UCM and 1% glycerol (*v*/*v*), followed by solid phase extraction of the sterile-filtered culture supernatant via an HLB cartridge, and chromatographic purification of the 50% MeOH (*v*/*v*) extract via a semi-preparative C_18_ column using UHPLC–ESI–MS. The strain label MS2 was used for *Roseovarius* sp. in the scheme (**b**) Total Ion Current (TIC, *m*/*z* 100–1,000, black) and UV spectrum (290 nm, red) of the semi-preparatively purified fractions via UHPLC. Of the 20 manually collected fractions, fraction 14 contained the *Roseovarius* factor. (**c**) Mass spectrometry of active fraction 14 (retention time = 14.31 min) indicates a mixture of unidentified *m/z* features. (**c**, **d**) Bioactivity assay of the active fraction 14 (green frame) and fractions 12, 13 and 15 as representative examples of negative results : Comparison with the control samples—axenic *Ulva* and *Ulva* cultured with *Roseovarius* sp. —revealed that fraction 14 exhibits *Roseovarius*-specific activity. In contrast, fractions 12, 13 and 15, representative of all other inactive fractions, showed no activity, resulting in the formation of axenic cell clusters

For further purification, the 50% methanol Oasis HLB extract was subjected to bioassay-guided chromatographic fractionation on a semi-preparative C18 column (Fig. 6a), yielding 20 fractions (Fig. 6b). Total ion chromatograms (TIC, *m/z* 100–1,000) and UV spectra at 290 nm were recorded simultaneously during separation. Bioassay screening determined fraction 14 as containing the *Roseovarius* factor (Fig. 6c). This fraction displayed strong mass spectrometric signals; however, the high matrix complexity and abundance of MS peaks precluded definitive identification of the active compound(s).

## Discussion

Our study focused on screening marine *Rhodobacterales* (*Alphaproteobacteria*) strains to evaluate the prevalence of strains that release bioactive factors phenocopying *Roseovarius* sp. MS2 and the partial purification of these compounds. A high prevalence of *Roseovarius*-like morphogenetic activity was observed within the order *Rhodobacerales*. Building on the observation by Grueneberg et al. (2016) that bioactive factors phenocopying *Roseovarius* sp. MS2 occur across α- and γ-proteobacteria, we systematically screened additional 96 genome-sequenced, well-growing and phylogenetically diverse *Rhodobacterales* strains from the DSMZ culture collection.

Notably, 79% of the 96 tested strains could substitute *Roseovarius* sp. MS2 in the tripartite community, inducing cell differentiation in *U. compressa*. They comprised strains from 11 different clades of the family *Roseobacteraceae* as well as 2 strains of clade 8, which represent the sister family *Paracoccaceae*. This widespread activity suggested that many bacteria, including the well-investigated genera *Roseovarius*,* Sulfitobacter* and *Rugeria* (Ghaderiardakani et al. 2017, 2024; Grueneberg et al. 2016; Ochiai and Goshima 2025), possess the capacity to synthesize morphogenetic compounds, suggesting that the underlying activity may be associated with conserved metabolic processes (Demain 1980).

In detail, some strains, including *Aliiroseovarius crassostreae*, *Aliiroseovarius halocynthiae*, and *Maritimibacter alkaliphilus*, clustered as non-inducers, suggesting a loss of morphogenetic activity. This finding may be attributable to the absence or pseudogenization of biosynthetic gene clusters, regulatory divergence, or specialization to distinct ecological niches. Supporting this, *Aliiroseovarius halocynthiae* was isolated from the sea squirt *Halocynthia roretzi* in the Damariscotta River (Maine, USA), and *A. crassostreae* from juvenile oyster disease–affected *Crassostrea virginica* in Namhae (South Korea) (Park et al. 2015). Their isolation from marine animals rather than algae is consistent with niche adaptation, which may reduce the selective pressure for morphogenetic interactions with macroalgae and favor alternative functional roles.

Comparative genome, transcriptome, and metabolome analyses could further clarify the genetic and evolutionary mechanisms behind this divergence, providing deeper insight into functional adaptation and selective pressures within *Rhodobacterales*. Future studies aimed at identifying the morphogenesis factor could focus on bacterial genera that include strains with and without morphogenetic activity, such as *Celeribacter* (Clade 7) and *Phaeobacter* (Clade 1). The most promising approach may be to compare the well-studied model organism *P. inhibens* DSM 17,395 (Frank et al. 2015; Thole et al. 2012) with the closely related type strain *P. gallaeciensis* DSM 26640^T^ (Buddruhs et al. 2013; Tomasch et al. 2025). The library of 5,500 transposons from *P. inhibens* DSM 17395 could be used to test *in sili*co predictions about genes involved in the biosynthesis of the morphogenesis factor (Smith et al. 2021). However, we cannot rule out that the absence of activity in strains like *P. gallaeciensis* might be a dosage effect, as suggested by the growth-phase dependence of the morphogenesis factor release in *Roseovarius* sp. MS2, which could complicate the identification of the underlying genes.

Geographically, inducing and non-inducing *Rhodobacterales* strains showed no clear spatial segregation in this data set (Fig. 2a, Table S1). The lack of a strong correlation between phylogenetic position and geographic origin suggests that functional traits related to *Ulva* morphogenesis are widely distributed, likely facilitated by the cosmopolitan distribution of both *Rhodobacterales* and *Ulva* species in coastal habitats. This finding can be applied to test the hypothesis that ecological interactions, rather than geographic isolation, are likely important drivers of the observed functional diversity. The global distribution of *Ulva*, which inhabits a wide range of marine environments (Tran et al. 2022) and releases photosynthates (Alsufyani et al. 2017), likely facilitates the worldwide presence of algal growth-inducing strains. However, a more systematic analysis including a larger and more evenly distributed set of strains (Tomasch et al. 2025) will be required to enable robust statistical evaluation of geographic and phylogenetic patterns.

Most strains originated from coastal and surface waters, with only a few from deep-sea environments (> 200 m). While *Celeribacter indicus* (2,946 m) and *Pseudophaeobacter arcticus* (167 m) lacked inducing activity, *Pseudooceanicola nanhaiensis* (~ 1,500 m) exhibited growth-promoting effects (Gu et al. 2007). These observations suggest that morphogenetic activity can occur in deep-sea lineages, albeit infrequently. The coexistence of inducing and non-inducing strains within similar niches indicates functional diversification across *Rhodobacterales* clades, potentially driven by niche specialization and adaptive trade-offs. The sporadic occurrence of inducing strains in deep-sea habitats suggests that morphogenetic activity may arise under specific ecological conditions, whereas its absence in other lineages may reflect shifts toward alternative strategies.

Overall, the tree illustrates that the phylogeny provides important context for predicting morphogenetic potential (Fig. 3), but that horizontal gene transfer, ecological adaptation, and possible gene-loss events may have contributed to the distribution of the AGMPF-trait within *Rhodobacterales*. Therefore, it is essential to integrate phylogenetic frameworks with phenotypic assays to comprehend the evolutionary and ecological dynamics of microbial-algal interactions.

Another key aspect of our study is the establishment of a bacteria-free system for research and industrial applications. The sterile-filtered bacterial culture supernatant effectively induced cell differentiation in *Ulva* and successfully replaced *Roseovarius* sp. MS2 in the tripartite system. This approach, to our knowledge, enables the cultivation for the first time of fully developed axenic *Ulva* spp. through the addition of sterile-filtered *Roseovarius* supernatant and thallusin. We provide a detailed protocol for preparing AUGM, enabling the cultivation of *Ulva* under strict axenic conditions without the need for symbiotic bacteria. This advancement provides a framework for applications that depend on axenic *Ulva* cultures. For instance, in pollutant degradation studies for bioremediation processes, axenic cultures enable the precise determination of whether degradation or removal from the growth medium is mediated by *Ulva* itself or its associated bacteria (Hardegen et al. 2023, 2025).

Moreover, axenic *Ulva* cultures are indispensable for high-resolution omics studies, including genomics, transcriptomics, and proteomics. These approaches aim to elucidate genetic communication in *Ulva*, where contamination by bacterial DNA, RNA, or proteins could otherwise compromise the accuracy of the results. Furthermore, axenic cultivation is critical for the isolation and characterization of bioactive compounds from *Ulva* for pharmaceutical and medical applications. The absence of foreign biotics enhances the purity and reproducibility of these extracts and simplifies regulatory and bureaucratic processes for the approval and commercialization of *Ulva*-derived pharmaceuticals, food products, and cosmetics (Toro-Mellado et al. 2025). Thus, our protocol provides a robust foundation for advancing research and industrial applications involving *Ulva* and addresses key challenges in ensuring experimental precision and regulatory compliance.

Furthermore, solid-phase extraction (SPE) was applied to gain insights into the physicochemical properties of the *Roseovarius* factor. Using cartridges with distinct binding properties (Oasis HLB, Chromabond EASY, Chromabond HILIC, and HyperSep Hypercarb), we assessed their polarity and charge. The Oasis HLB cartridge retained the bioactive compound, which was eluted with 50% methanol (v/v), suggesting that the active compound(s) exhibit moderate polarity and amphiphilic characteristics. This is consistent with the hydrophilic–lipophilic balance of the HLB polymer matrix, which is known to retain moderately polar compounds (Bäuerlein et al. 2012; Dias and Poole 2002). In contrast, the Chromabond EASY cartridge did not retain the activity, with bioactivity detected in the flow-through, indicating differences in binding efficiency likely due to polymer composition or interaction mechanisms. The HILIC cartridge failed to adsorb the compound, suggesting it is neither highly polar nor strongly charged (Ikegami et al. 2008). Similarly, the HyperSep Hypercarb cartridge, which preferentially retains highly nonpolar compounds, did not retain activity, confirming that the compound is not strongly hydrophobic (Hennion 2000). Together, these results constrain the physicochemical properties of the active compound(s) to a moderately polar range. In addition, non-retentive cartridges may be useful for removing matrix components prior to HLB-based enrichment.

The inferred amphiphilic nature of the *Roseovarius* factor is consistent with known microbial signaling molecules involved in symbiotic interactions, such as acyl-homoserine lactones (Churchill and Chen 2011). While other amphiphilic compounds (e.g., antimicrobial peptides or saponins) may share similar properties, their involvement in this context remains speculative. Subsequent fractionation using semi-preparative C_18_ chromatography further enriched the bioactive fraction (Decroo et al. 2017), demonstrating the effectiveness of the purification workflow for complex biological extracts and supporting previous findings on bioactive metabolites from marine bacteria (Imhoff et al. 2011).

In the characterization of bioactive fractions, we demonstrated that the algal growth-inducing activity of the bacteria-free *Roseovarius* culture supernatant (harvested at OD_620_ = 0.1) increases significantly with its relative concentration in AUGM (Fig. 4). This suggests that higher proportions of the supernatants in the culture medium promote dose-dependent *Ulva*´s growth. While AGMPFs are essential drivers of morphogenesis and development of *Ulva*, additional nutritional or metabolic factors can contribute to algal growth (Li et al. 2016; Mouget et al. 1995).

Intriguingly, the bioactivity of the undiluted *Roseovarius* sp. supernatant (100% *v*/*v*) depended on the presence of *Maribacter* sp. (Fig. 4b–e), with activity observed only in combination. A similar pattern occurred in selected C_18_ fractions (data not shown). This may reflect the accumulation of inhibitory metabolites at high concentrations, which may subsequently be modified or complemented by *Maribacter* sp., resulting in distinct morphogenetic outcomes. Such interactions are consistent with co-metabolism and cross-feeding within microbial consortia (Ponomarova and Patil 2015; Wienhausen et al. 2024). The tripartite *Ulva–Roseovarius–Maribacter* system thus illustrates the complexity of microbial–algal symbioses. *In natural* environments or under standard culture conditions, *Ulva* can control the beneficial and inhibitory effects of *Roseovarius* sp. by regulating glycerol availability and thereby controlling bacterial growth (Kessler et al. 2018).

The restoration of growth upon dilution indicates a concentration-dependent balance between inhibitory and growth-promoting compounds. While inhibitory effects dominate at high concentrations, AGMPFs may be produced at higher levels during early exponential growth or remain active at low concentrations, as observed for thallusin (Dhiman et al. 2022). This highlights the importance of production dynamics and activity thresholds in shaping the *Roseovarius–Ulva* interaction. These observations are also relevant for purification strategies, as dilution may restore activity in inhibitory extracts. Understanding this balance will be critical for optimizing purification workflows and maintaining AGMPF activity in downstream applications.

In conclusion, our study highlights the potential of macroalgae-associated bacteria, particularly within marine *Rhodobacterales*, as a source of growth-promoting factors in *Ulva*. Through systematic screening and targeted purification, we enriched bioactive fractions containing AGMPF activity that induces *Ulva* cell division, thereby advancing our understanding of microbial–algal interactions and enabling new approaches for axenic cultivation and the discovery of bioactive compounds. Despite successful enrichment, the chemical identity of the active compound(s) remains unresolved due to their low abundance and the matrix’s complexity. Co-extracted components interfered with mass spectrometric analysis, preventing the determination of molecular weight and functional groups. Even after repeated fractionation, the amount of purified material was insufficient for structural elucidation. Optimizing extraction and purification protocols to improve yield will support comprehensive structural elucidation by NMR and mass spectrometry.

Future work will focus on scale-up and advanced purification to enable structural elucidation and the development of quantitative bioassays in the presence and absence of *Maribacter* sp. MS6 and other *Ulva*-associated bacteria. Investigating the functional synergy between *Roseovarius* and *Maribacter* may reveal additional layers of bioactivity in marine microbial consortia. Previous studies showed that *Maribacter* sp. MS6 growth is stimulated by *Roseovarius* sp. MS2 (Spoerner et al. 2012), suggesting metabolic or signaling interactions, potentially mediated by released metabolites or environmental modifications. These observations highlight the cooperative dynamics within the tripartite community and the importance of both macroalgal–bacterial and bacterial–bacterial interactions. Integrating metabolomic and transcriptomic approaches in axenic systems treated with AGMPFs will further elucidate bacterial contributions to *Ulva* development and support the design of targeted consortia for aquaculture and biotechnology applications.

## Supplementary Information

Below is the link to the electronic supplementary material.

Supplementary Material 1 (CSV 18.3 KB)

Supplementary Material 2 (PDF 566 KB)

## Data Availability

Sequence data that support the findings of this study have been deposited in the National Center for Biotechnology Information with the accession codes in Genbank JBKHVF000000000.1, assembly: GCA_051447875.1, and BioProject PRJNA828511. Moreover, data is provided within the manuscript or supplementary information files.
