# Supplementary material for: Marine Rhodobacterales as Drivers of Ulva Growth: From Macroalgal–Bacterial Interactions to Bioactive Factor Enrichment
Source: J Chem Ecol. 2026 May 19;52(3):45. doi: 10.1007/s10886-026-01720-8 (PMC13186880; doi:10.1007/s10886-026-01720-8)
Supplement: Supplementary file 2 — Supplementary Material 2 (PDF 566 KB) [file 10886_2026_1720_MOESM2_ESM.pdf]

Supporting Information for

Marine Roseobacteraceae as Drivers of *Ulva* Growth: From macroalgal-bacterial Interactions to Bioactive Factor Enrichment

Johann F. Ulrich[1], Simon B. Redlich[1], Anne Mohr[1], John Vollmers[2], Jörn Petersen[3,4], Thomas Wichard[1*]

1-Institute for Inorganic and Analytical Chemistry, Friedrich Schiller University Jena, Lessingstr. 8, D-07743 Jena, Germany

2-Institute for Biological Interfaces 5, Karlsruhe Institute of Technology, Karlsruhe, Germany

3-Leibniz Institute DSMZ – German Collection of Microorganisms and Cell Cultures, Braunschweig, Germany

4-Institute of Microbiology, Technical University of Braunschweig, Braunschweig, Germany

* Correspondence: thomas.wichard@uni-jena.de; Tel.: +49-3641-948184

Contents

Fig. S1: Bioassay. Standardized morphogenetic bioassay with *Ulva compressa*....................... 3

Fig. S2: *Ulva compressa* morphogenetic bioassay screening with selected bacterial strains showing novel morphotypes.. 4

Fig. S3: Dose-dependent effect of *Roseovarius* sp. MS2 supernatant on longitudinal growth of *Ulva compressa*. 5

Fig. S4: Morphogenesis-guided *Ulva* bioassays to characterize the thermal and UV stability of the *Roseovarius* factor. 6

Figure S1

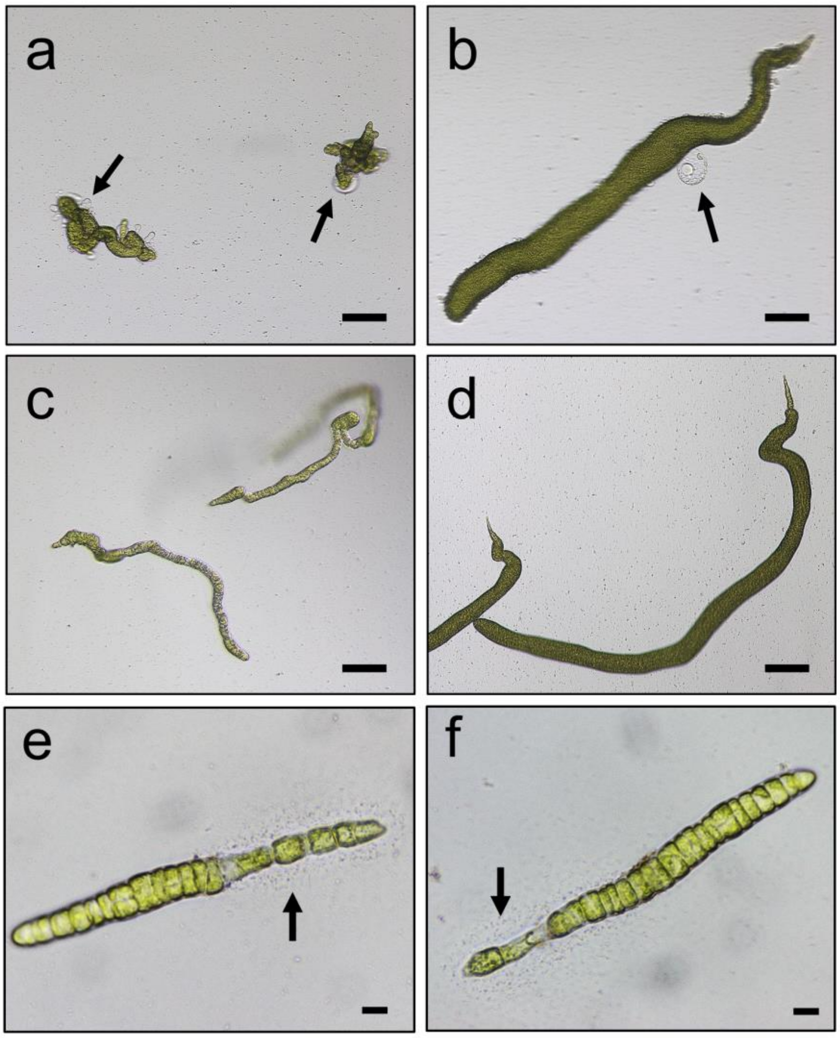

Fig. S1: Bioassay. Standardized morphogenetic bioassay with *Ulva compressa*. Representative morphogenetic phenotypes of *U. compressa* were monitored after 2 weeks of cultivation, magnification bar = 100 µm: (a) axenic cultures with undefined cell aggregations and cell wall protrusions (cell wall protrusions, black arrow); (b) *Roseovarius* activity with typical thallus differentiation but damaged cell walls (cell wall protrusions, black arrow); (c) activity of *Maribacter* or thallusin, with rhizoid and cell wall formation (no cell wall protrusions); (d) tripartite community of *Ulva–Roseovarius–Maribacter* with complete morphogenetic growth; (e, f) representative examples for the accumulation of bacteria in the rhizoidal zone of 1 week old germlings (black arrow), scale bar = 10 µm. Note: Future studies should assess whether Rhodobacterales or other bacterial taxa can produce all AGMPFs required for *Ulva* development, thereby functionally replacing thallusin-producing strains such as *Maribacter* sp. MS6 as well.

Figure S2

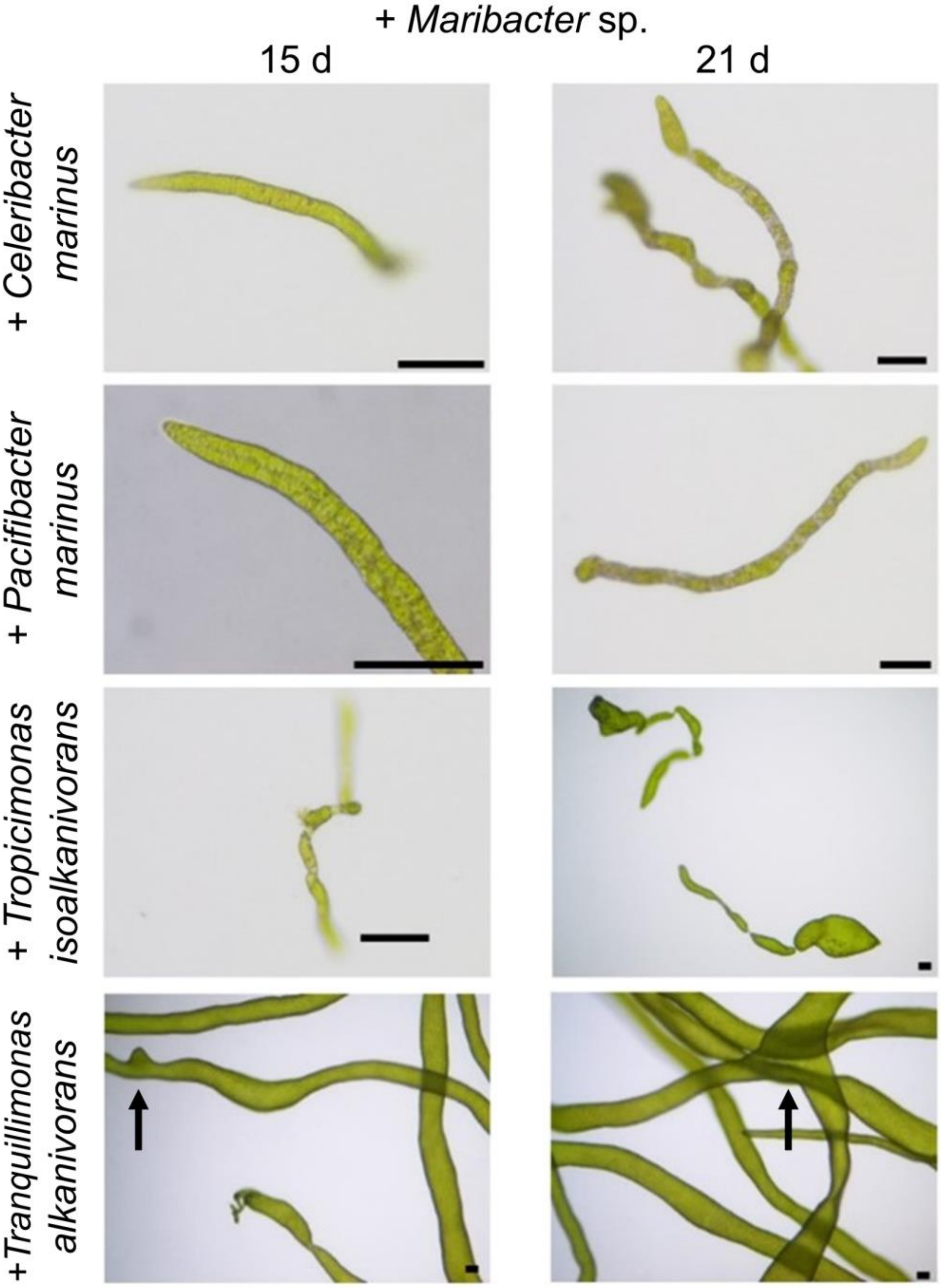

Fig. S2: ***Ulva compressa*** **morphogenetic bioassay screening with selected bacterial strains showing novel morphotypes**. The combination of the thallusin-producing *Maribacter* sp. MS6 with the respective test strains resulted in either algicidal effects or delayed development after 2–3 weeks of cultivation. Inoculation with *Tranquillimonas alkanivorans*, for instance, led to additional branching during thallus development (black arrow). Scale bar = 100 µm.

Figure S3

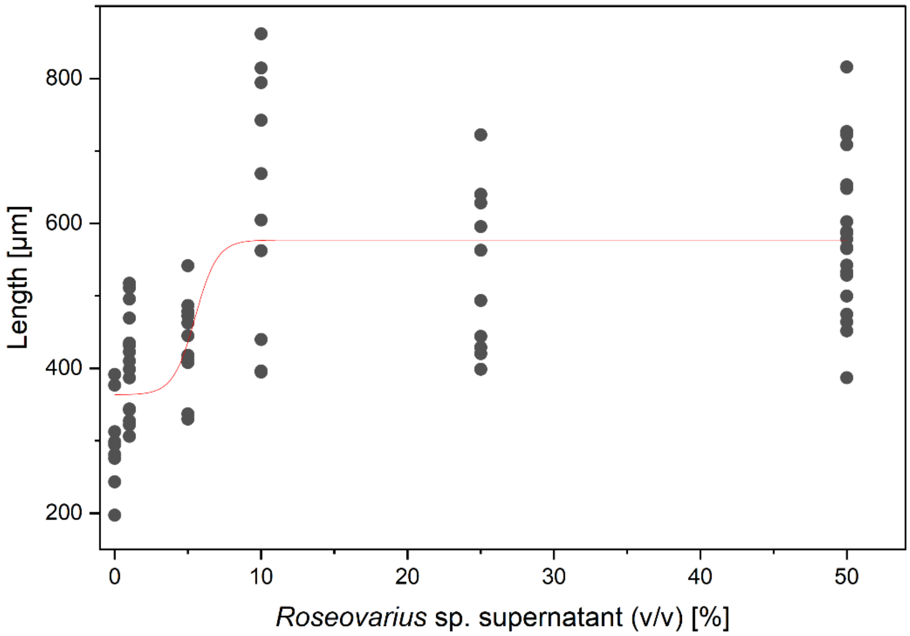

Fig. S3: Dose-dependent effect of *Roseovarius* sp. MS2 supernatant on longitudinal growth of *Ulva compressa*. Individual data represent algal length measurements after 14 days of cultivation at increasing proportions of sterile-filtered supernatant (*v*/*v*). Growth increased with rising supernatant concentrations and approached saturation at ~10% (v/v). The red line indicates a fitted dose–response curve showing a plateau in growth-promoting activity at higher concentrations. The half-maximal effective concentration (EC_{50}) was determined to be 5.5%.

Figure S4

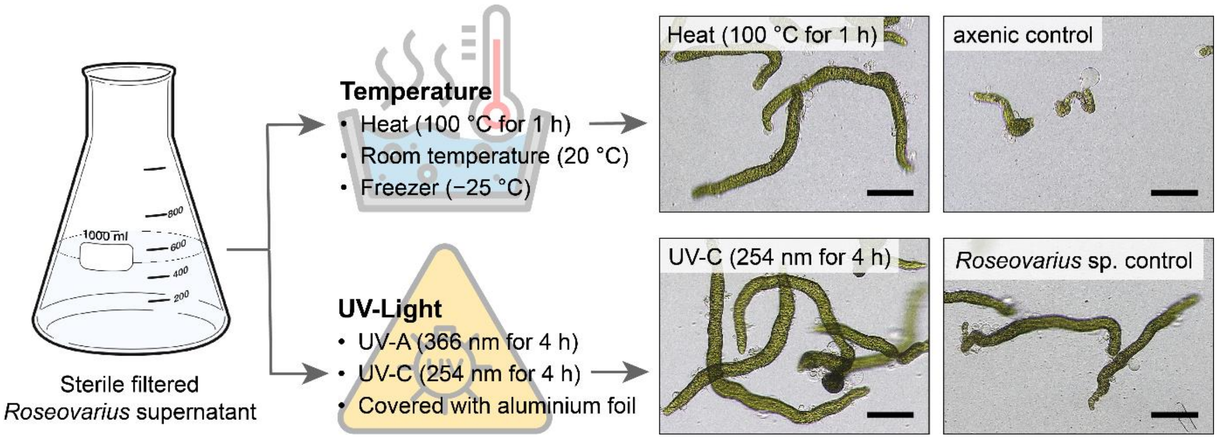

Fig. S4: Morphogenesis-guided *Ulva* bioassays to characterize the thermal and UV stability of the *Roseovarius* factor. The sterile-filtered *Roseovarius* supernatant was subjected to various physical treatments to assess its stability. To test thermostability, the supernatant was boiled in a water bath at 100 °C for one hour. As a reference and negative control, one sample was maintained at room temperature (20 °C), and another was frozen at −25 °C. The supernatant was exposed to UV-C (254 nm) for 4 hours to assess UV stability, and UV-A (366 nm) was also tested. The negative control was kept in the dark, covered with aluminum foil. All treated samples were tested in *Ulva* bioassays and retained their biological activity. The *Ulva* cultures were grown for 14 days in microwell plates. Scale bar = 100 µm.
